# Supplementary material for: Changes in Physical Activity during the COVID-19 Pandemic—An Analysis of Differences Based on Mitigation Policies and Incidence Values in the Federal States of Germany
Source: Sports (Basel). 2021 Jul 15;9(7):102. doi: 10.3390/sports9070102 (PMC8309874; doi:10.3390/sports9070102)
Supplement: Supplementary file 1 [file sports-09-00102-s001.zip › Supplementary Material Table S1.pdf]

Supplementary Material Table S1 Policy index for each federal state

Federal state (mean 7-day-incidence value / 100.000 inhabitants)	Large events with a maximum of xxx people<br><br>1= ≥ 1,000<br>2= 500-999<br>3= 200-499<br>4= max. 200	Private celebrations with a maximum of xxx people<br><br>1= > 50<br>2= 30-50<br>3=11-30<br>4= max. 10	Meetings in public space with a maximum of xxx people<br><br>1= > 15<br>2= 11-15<br>3= 6-10<br>4= max. 5	Amount of fine not wearing a mouth-nose-covering<br><br>1= no fine<br>2= max. 50 EUR<br>3= 50-150 EUR<br>4= > 150 EUR	Schools are…<br><br>1= normally open<br>2= opened under hygiene requirements<br>3= alternate lessons<br>4= closed	Allowed number of people in organized sports<br><br>1= no restriction<br>2= > 60<br>3= 31-60<br>4= up to 30	Square meters (sqm) per person in retail<br><br>1= no restriction<br>2= 1.5m<br>3= ≥ 10sqm<br>4= ≥ 11sqm	Index
Baden-Wuerttemberg (46.7)	4	4	3	3	2	4	2	22
Bavaria (48.2)	3 outdoors and indoors with certain permanent seating<br>4 Indoors and outdoors without permanent seating<br>= 3.5	4	3	4	2	4	3	23.5
Berlin (85.2)	1	3 outdoors<br>4 indoors<br>= 3.5	4	3	2	4	2	19.5
Brandenburg (21.8)	1	3	3	3	2	1	2	15
Bremen (80.4)	3	3	3	2	2	3	2	18
Hamburg (43.4)	1 outdoors with permanent seating<br>2 indoors with permanent seating<br>4 outdoors and indoors without permanent seating<br>= 2.3	3	3	3	2	4	3 with retail space 0-800 sqm<br>4 with retail space > 800 sqm<br>= 3.5	20.8
Hesse (61.3)	3	2	3	2	2	1	2	15

Lower Saxony (31)	2	2 outdoors<br>3 in own flat<br>1 private celebrations in public premises<br><br>= 2	3	4	2	3	2	18
Mecklenburg Vorp. (15.5)	2 outdoors<br>4 indoors<br>= 3	1	3	3	2	2	3	17
Northrhine-Westphalia (60.5)	1	3	3	3	2	1	3	16
Rhineland Palatinate (37)	2 outdoors<br>3 indoors<br>= 2.5	3	3	2	2	4	3	19.5
Saarland (54.7)	3 outdoors<br>4 indoors<br>= 3.5	3 indoors<br>4 indoors in private premises<br>= 3.5	3	3	2	3	4	22
Saxony (37.3)	1	3	3	3	1	1	2	14
Saxony-Anhalt (15.1)	1 outdoors<br>2 indoors<br>= 1.5	2	3	1	1	3	2	13.5
Schleswig Holstein (18.8)	1 outdoors with permanent seating<br>2 indoors with permanent seating<br>4 outdoors and indoors without permanent seating<br><br>= 2.3	2	3	3	2	1	2	15.3
Thuringia (22.3)	1	1 outdoors<br>2 indoors<br>= 1.5	3	3	2	1	1	12.5
