# Supplementary material for: Changes in Physical Activity during the COVID-19 Pandemic—An Analysis of Differences Based on Mitigation Policies and Incidence Values in the Federal States of Germany
Source: Sports (Basel). 2021 Jul 15;9(7):102. doi: 10.3390/sports9070102 (PMC8309874; doi:10.3390/sports9070102)
Supplement: Supplementary file 1 [file sports-09-00102-s001.zip › Supplementary Material Figure S1.pdf]

Supplementary Material Figure S1: PA changes for each federal state

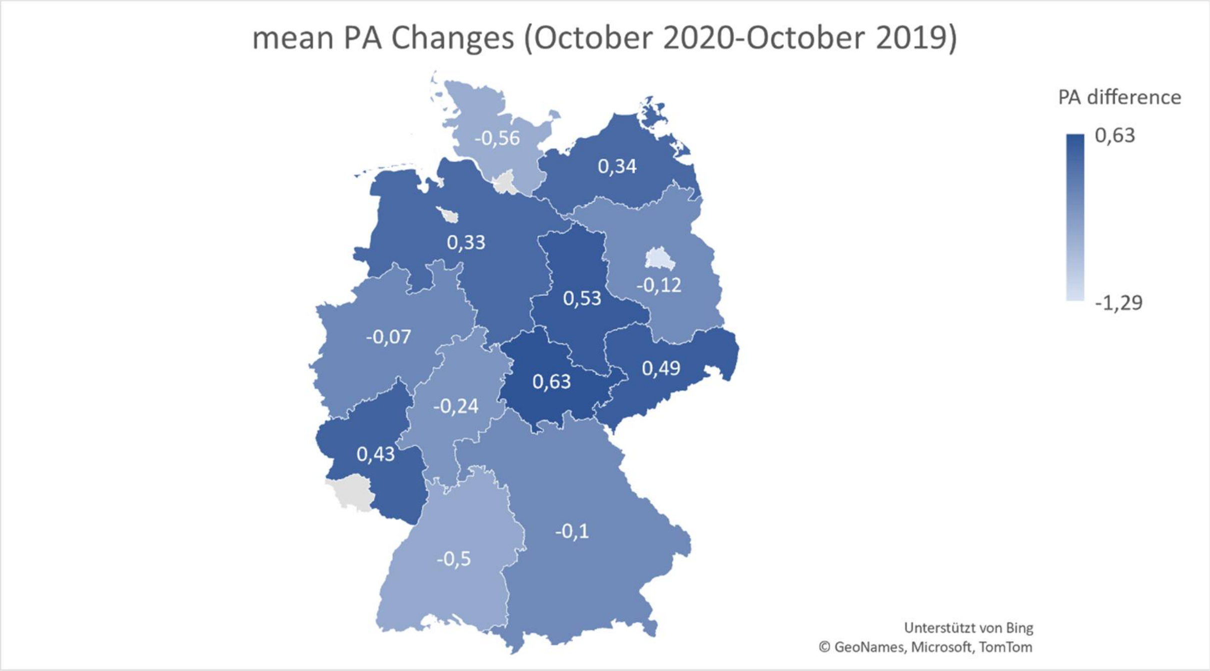

Mean change in PA (hours per week) between pre-Covid-19 and October 2020 in each federal state. A positive value indicates an increase whereas a negative value indicates a decrease of PA.
